# Prenatal nicotine exposure alters gene expression profiles of neurons in the sub-regions of the VTA during early postnatal development

**DOI:** 10.1038/s41598-023-31997-8

**Published:** 2023-03-25

**Authors:** Lindsey D. McGill, Naze G. Avci, Tina Kazemi, Yoshinori Sunaga, Yasemin M. Akay, Metin Akay

**Affiliations:** Department of Biomedical Engineering, University of Houston, Houston, TX 77204 USA

**Keywords:** Developmental biology, Neuroscience, Reward

## Abstract

Brain growth occurs during the first 2 weeks of postnatal development in rats. This developmental period is equivalent to the third trimester of human gestation. Dendritic arborization, axonal growth, and gliogenesis are observed along with a strong maturation of neurotransmission during this critical development period. Furthermore, nicotine exposure during early development causes deficiencies in sensory and cognitive processing in adults. In this study, we further investigated the gene expression of neuron groups and the influence of perinatal nicotine exposure on gene expressions of neurons within the sub-regions of the ventral tegmental area (VTA) in 1 week, 2 week and 3-week-old rat pups. We exposed pregnant rats to nicotine perinatally on gestational day 7 through postnatal day 14. Pups are exposed to nicotine during pregnancy and through breastfeeding to investigate its effect in rat pups during early neuronal development. Real time PCR was used to find the relative expressions of gamma-aminobutyric acid (GABA), dopamine, and glutamate neuron markers within the three sub-regions of the VTA including the parabrachial pigmented nucleus (PBP), parainterfascicular (PIF), and paranigral nucleus (PN). Our results indicated that during early maturation, the dopamine marker tyrosine hydroxylase (TH) showed a consistently increased significance in PN sub-region compared to PIF and PBP. These results suggest that following perinatal nicotine exposure, VTA dopamine neurons, especially within the PN sub-region, are significantly excited starting from birth.

## Introduction

Maternal smoking and nicotine usage are associated with higher rates of stillbirth, sudden infant death syndrome (SIDS), attention-deficit/hyperactivity disorder (ADHD), adverse effects on cognitive and behavioral development in children, and an increased risk of developing a dependency on nicotine later in the life of the offspring^1^. On average, 15–25% of women smoke during pregnancy, and millions of pregnant women engage in smokeless tobacco use^1^. Furthermore, 25% of children are exposed to nicotine during gestation in industrialized countries^2^. Because nicotine can be passed to the offspring through the placenta, nicotine concentration levels exceeding maternal concentrations by 15% in fetal circulation and a concentration 88% higher than maternal plasma in amniotic fluid was detected in prenatal humans^1^. Additionally, nicotine accumulates in breast milk and has been shown to accumulate in the fetal brains of rats^3^. We have previously conducted gene expression studies on many samples for each experimental group using microarray and qPCR method with consistent results^4,5^ These findings suggest that prenatal nicotine exposure affects brain development as nicotine receptors are present in the first trimester^6^. The motor, sensory, and cognitive deficits observed in infants associated with in-utero exposure to tobacco also suggest that nicotine affects early neurodevelopment^1,2^.

The ventral tegmental area (VTA) of the midbrain is associated with reward and drug addiction that plays a crucial role in the release of dopamine and GABA, and its circuitry can be altered when exposed to addictive drugs or behavior^7^. Neurons of the VTA consist of 60–65% dopaminergic (DA), 35% γ-aminobutyric acid (GABA)ergic, and glutamatergic neurons^8^. The VTA consists of three sub-regions: the parabrachial pigmented nucleus (PBP), parainterfascicular nucleus (PIF), and paranigral nucleus (PN)^9^. Specifically, nicotine use targets the VTA where dopamine is released to the nucleus accumbens (NAc)^1^. In addition to the dopaminergic pathways, the VTA DA neurons are controlled by glutamatergic synaptic inputs from the prefrontal cortex (PFC)^10^. Previously, in our lab, we have shown that exposure to nicotine increases DA neuron activity in the VTA, which increases neuronal firing complexity between the VTA and the PFC^9^. Interactions with addictive drugs have shown behaviors of aggression, compulsion, and lack of social play because of the mesocorticolimbic DA circuitry with the NAc and PFC^11^. Previously, our lab published a study that observed the three sub-regions of the VTA in rats following gestational exposure to nicotine at 28 days after birth^12^. Samples were collected from 28-day old rat pups following exposure to nicotine prenatally as well as through breast milk after birth^12^. Gene expression from these three sub-regions sought to show the expression of GABA (GAD1, also known as GAD67, and GAD2, also known as GAD65), DA (Slc6a3, TH), and glutamate (Slc17a6) neuron markers when nicotine-exposed pups were compared with saline exposed pups^12^. It was most notable that DA neurons are more activated in the PN sub-region of the VTA. Our current study investigates gene expression of neuron groups in the sub-regions of the VTA and gene expressions to gain insight into the dopaminergic development of the VTA in rat pups following perinatal exposure to nicotine at 7 (P7), 14 (P14), and 21 (P21) days after birth since most neuronal cell groups and early synaptogenesis occurs during this developmental stage^13–15^. To examine the expression levels of neurons within the sub-regions of the VTA, we tested specific gene expressions in rat neurons exposed perinatally with nicotine compared to rat neurons exposed perinatally with saline. Real time PCR (qPCR) was used to find the relative expression of GABA, DA, and glutamate neuron markers.

## Results

### Relative gene expressions in the sub-regions of the VTA

**Figure 1:**
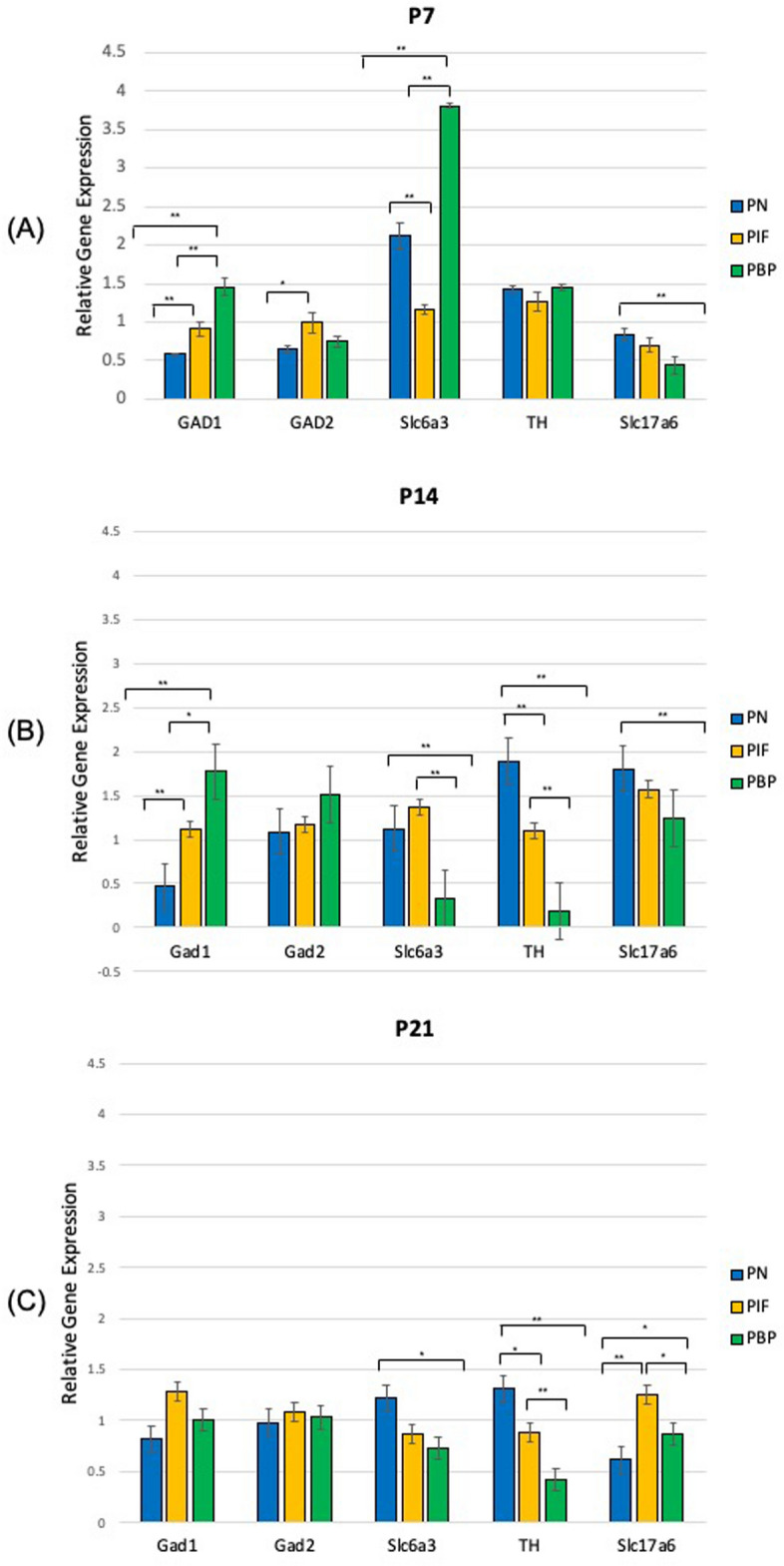
Relative gene expression of GABA, DA, and glutamate markers by sub-region of the VTA following nicotine exposure as compared to the saline group. The results follow qPCR analysis relative to housekeeping gene, GAPDH. Two-way analysis of variance was used to indicate the significance between gene expressions in each sub-regions of the VTA as well as each gene for (**A**) age P7 (nicotine: n = 7; saline: n = 7) (**B**) age P14 (nicotine: n = 20; saline: n = 14), and (**C**) age P21 (nicotine: n = 18; saline: n = 14). *p < 0.05, **p < 0.01, ± standard error.

We have previously conducted studies of gene expression using the qPCR method with a larger number of samples for each compared experimental group with consistent results^4,5^. In this study, to understand the neuron distribution and development of DA, GABA, and glutamate neurons following prenatal nicotine exposure in each sub-region of the VTA, samples were taken 7 (P7), 14 (P14) and 21 (P21) days after birth from nicotine exposed pups and were compared with saline groups of the same age. Samples were obtained by individual brain slices from each sub-region, and their relative gene expression was analyzed using the primers for qPCR as listed in Table 1. Results from the relative gene expression analysis using Comparative Ct Method (ΔΔCt) are shown in Fig. 1 and their significance was determined using the t-test method.

**Table 1:** List of primer sequences for GABA, DA, and glutamate neural markers.

Target gene	Primer sequence	Category	Assay ID	Lot number
Glutamate decarboxylase 1 (GAD1)	CAACCTGTTTGCTCAAGATCTGCTT	GABA	Rn00690300_m1	865386
Glutamate decarboxylase 2 (GAD2)	CGATTAAAACAGGGCATCCCCGATA	GABA	Rn00561244_m1	1804450
Solute carrier family 6, dopamine transporter (Slc6a3)	GTGGCCACAGATGGACCTGGGCTCA	DA	Rn00562224_m1	1881650
Tyrosine hydroxylase (TH)	CAAGGACAAGCTCAGGAACTATGCC	DA	Rn00562500_m1	1865386
Solute carrier family 17, glutamate transporter (Slc17a6)	GGACAGATCTACAGGGTGCTGGAGA	Glutamate	Rn00584780_m1	1764173
Glyceraldehyde-3-phosphate dehydrogenase (GAPDH)	AGGAGTCCCCATCCCAACTCAGCCC	Housekeeping Gene	Rn01775763_g1	1894565

In Fig. 1A, at P7, GAD1 showed a higher expression in the PBP sub-region compared to PN and PIF sub-regions (p < 0.01). GAD2 showed a higher expression in the PIF sub-region at P7 when compared to PN sub-region. Slc6a3 showed a significantly higher expression in PBP vs. PIF (p < 0.01) and PN vs. PIF (p < 0.01). Slc6a3 also showed a higher expression in the PBP sub-region when compared to PN sub-region (p < 0.01). We did not observe a significant change in TH expression in the VTA sub-regions. SLC17a6 showed a higher expression in PN vs. PBP sub-region (p < 0.01).

In Fig. 1B, at age P14, GAD1 showed a higher expression in the PBP sub-region when compared to PN and PIF sub-regions (p < 0.01 and p < 0.05, respectively). GAD1 showed a higher expression in the PIF sub-region when compared to PN sub-region (p < 0.01) at P14. We did not observe a significant change in GAD2 expression in the VTA sub-regions. Slc6a3 showed a higher expression in the PN sub-region when compared to PBP sub-region (p < 0.01). Slc6a3 also showed a higher expression in the PIF sub-region when compared to PBP sub-region (p < 0.01). Our results suggested that in PN and PIF sub-regions of the VTA, an increased expression of TH compared to the PBP sub-region was observed and was statistically significant (p < 0.01). The PN sub-region of TH group also showed a higher expression when compared to PIF sub-region (p < 0.01). Slc17ab showed a higher expression in the PN sub-region when compared to PBP sub-region (p < 0.01).

At P21, shown in Fig. 1C, GAD1 and GAD2 did not show a change in their expressions in any of the sub-regions. Slc6a3 showed a higher expression in the PN sub-region when compared to the PBP sub-region (p < 0.05). TH expression was higher in PN compared to both PIF and PBP (p < 0.05 and p < 0.01, respectively). TH showed a higher expression in the PIF sub-region when compared to PBP sub-region (p < 0.01). Slc17a6 expression was higher in PIF compared to PN and PBP (p < 0.01 and p < 0.05, respectively). Slc17a6 showed a higher expression in PBP sub-region when compared to PN sub-region (p < 0.05).

## Relative gene expressions in the sub-regions of the VTA compared to nicotine treated whole midbrain VTA samples

### P7 group

**Figure 2:**
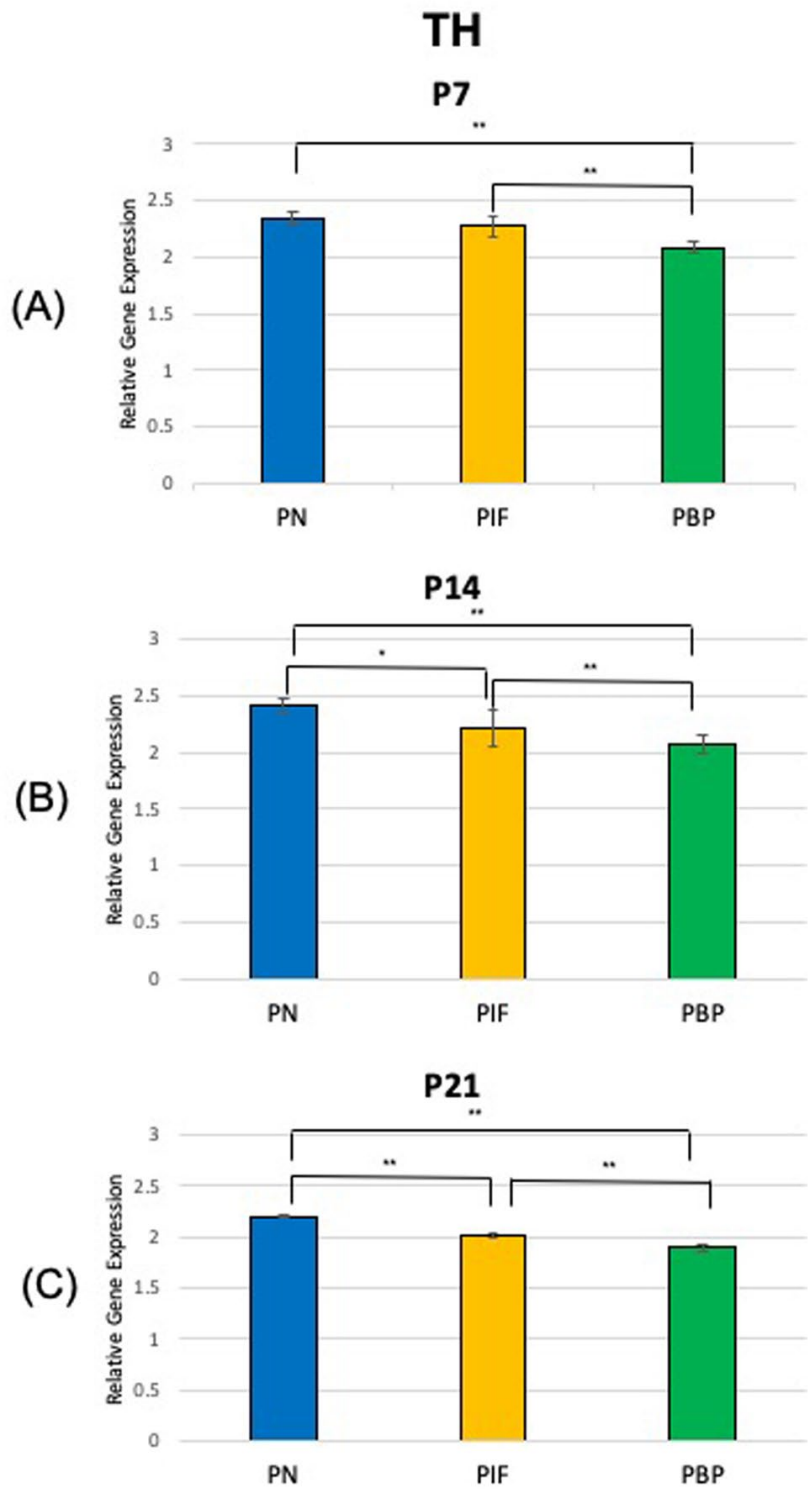
Relative gene expression of TH in the VTA sub-regions (PN, PIF, and PBP) of nicotine exposed pups normalized to nicotine treated whole VTA midbrain samples. qPCR was used to identify the expression levels of DA neurons for the (**A**) P7, (**B**) P14, and (**C**) P21 day old rat pups. ***p < 0.001, standard deviation. **p < 0.01, ± standard deviation. *p < 0.05 ± standard deviation.

**Figure 3:**
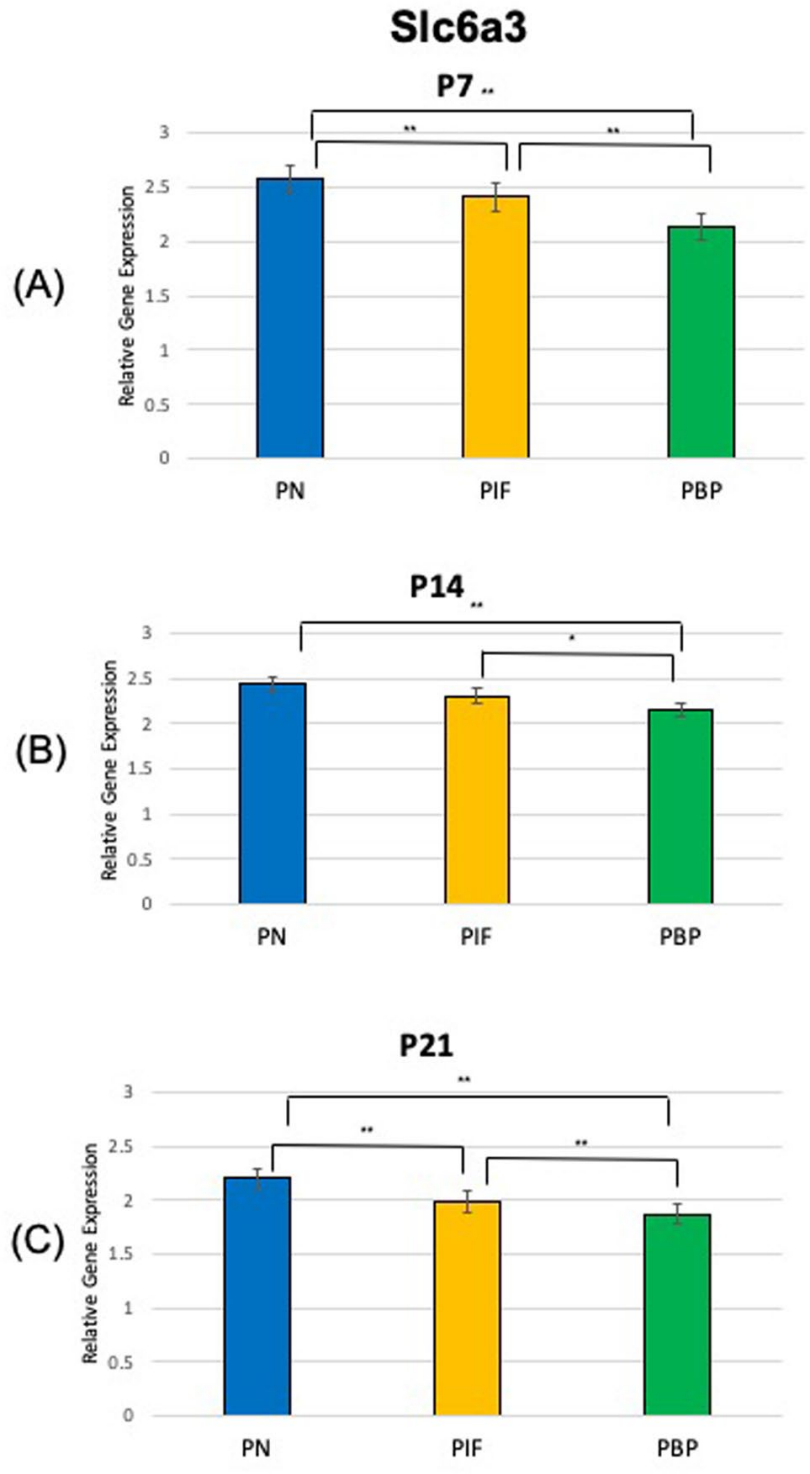
Relative gene expression of Slc6a3 in the VTA sub-regions (PN, PIF, and PBP) of nicotine exposed pups normalized to nicotine treated whole VTA midbrain samples. qPCR was used to identify the expression levels of DA neurons for the (**A**) P7, (**B**) P14, and (**C**) P21 day old rat pups. ***p < 0.001, standard deviation. **p < 0.01, ± standard deviation. *p < 0.05 ± standard deviation.

**Figure 4:**
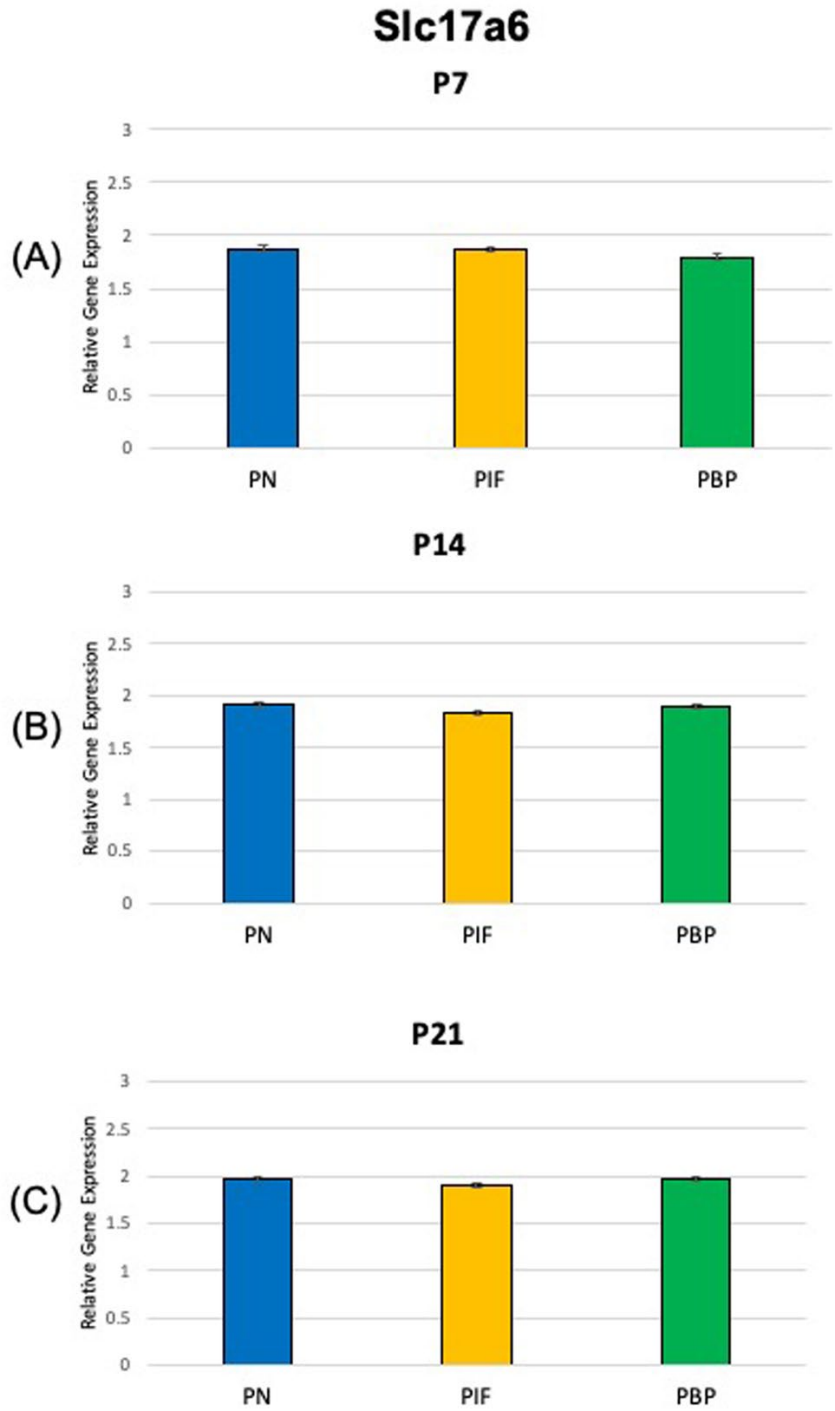
Relative gene expression of Slc17a6 in the VTA sub-regions (PN, PIF, and PBP) of nicotine exposed pups normalized to nicotine treated whole VTA midbrain samples. qPCR was used to identify the expression levels of glutamate neurons for the (**A**) P7, (**B**) P14, and (**C**) P21 day old rat pups. ***p < 0.001, standard deviation. **p < 0.01, ± standard deviation. *p < 0.05 ± standard deviation.

**Figure 5:**
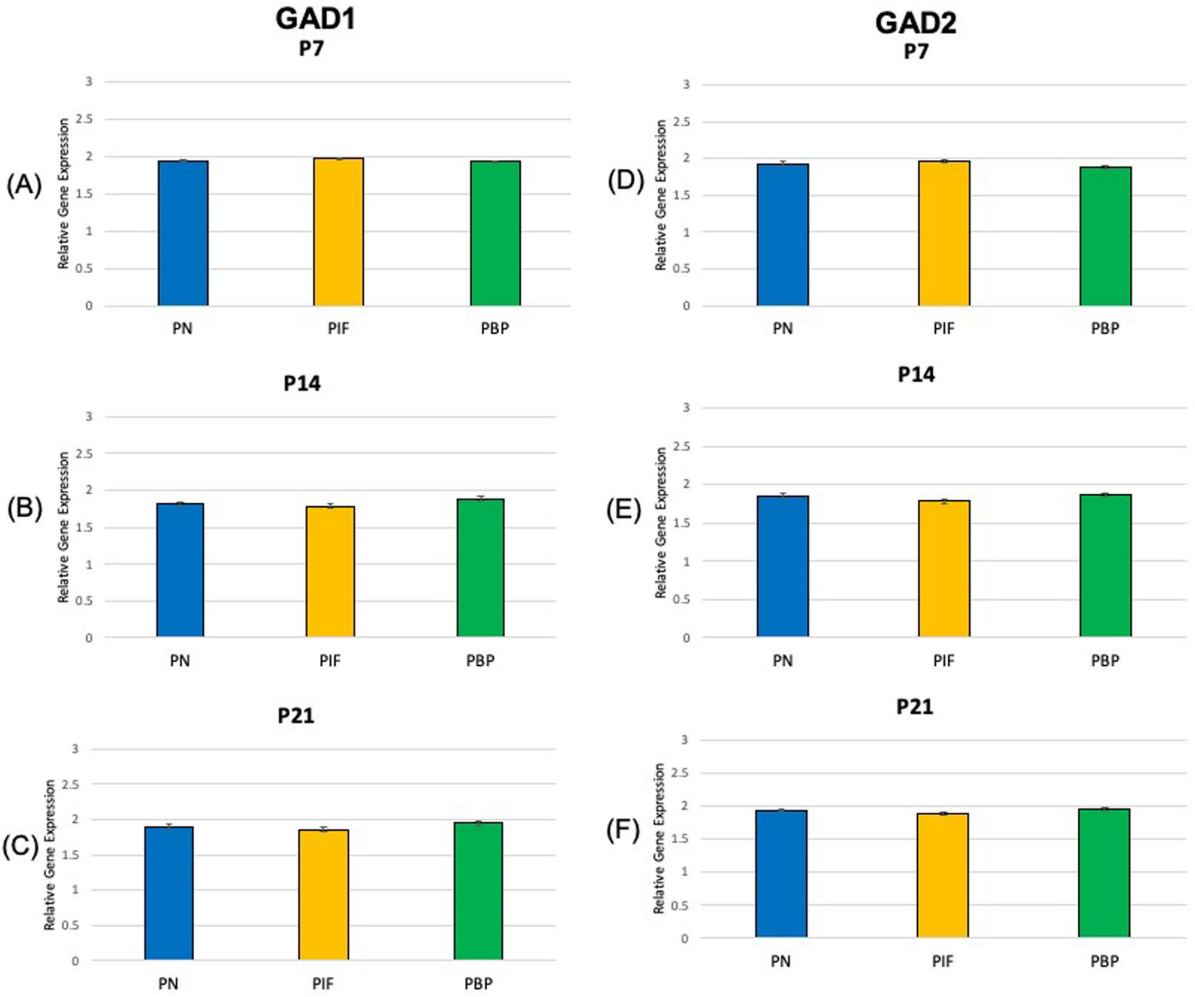
Relative gene expression of GAD1 and GAD2 in the VTA sub-regions (PN, PIF, and PBP) of nicotine exposed pups normalized to nicotine treated whole VTA midbrain samples. qPCR was used to identify the expression levels of GABA neurons for the (**A**) GAD1 P7, (**B**) GAD1 P14, (**C**) GAD1 P21, (**D**) GAD2 P7, (**E**) GAD2 P14, and (**F**) GAD2 P21 day old rat pups. ***p < 0.001, standard deviation. **p < 0.01, ± standard deviation. *p < 0.05 ± standard deviation.

At age P7, our results suggested TH showed a significantly higher expression in the PN and PIF sub-regions when compared to the PBP sub-region (p < 0.01) of nicotine exposed pups when normalized to nicotine treated whole VTA midbrain samples. No significance was observed between PN and PIF sub-region (Fig. 2A). Slc6a3 expression was higher in PN sub-region compared to PIF and PBP subregions (p < 0.01). Slc6a3 expression was higher in PIF sub-region compared to PBP subregion (p < 0.01) (Fig. 3A). Slc17a6 did not show a change in different sub-regions as shown in Fig. 4A. As shown in Fig. 5A,D, GAD1 and GAD2 did not show a change in their expressions in any of the sub-regions.

### P14 group

At age P14, TH showed a significantly higher expression in the PN when compared to both PIF and PBP sub-regions (p < 0.05 and p < 0.01, respectively) (Fig. 2B). TH showed a higher expression in the PIF sub-region when compared to the PBP sub-region (p < 0.01) (Fig. 2B). Slc6a3 expression was higher in PN sub-region compared to PBP sub-region (p < 0.01). Slc6a3 expression was higher in PIF sub-region compared to PBP sub-region (p < 0.05). No significance was observed between PN and PIF sub-region (Fig. 3B). Slc17a6 did not show a change in different sub-regions as shown in Fig. 4B. As shown in Fig. 5B,E, GAD1 and GAD2 did not show a change in their expressions in any of the sub-regions.

### P21 group

At age P21, we observed that TH showed a significantly higher expression in the PN sub-region when compared to both PIF and PBP sub-regions (p < 0.01) (Fig. 2C). This change in PN sub-region was consistently observed throughout the study at P7 and P14 (Fig. 2A,B). TH showed a higher expression in the PIF sub-region when compared to the PBP sub-region (p < 0.01). Slc6a3 expression was higher in PN sub-region compared to both PIF and PBP sub-regions (p < 0.01) (Fig. 3C). Slc6a3 showed a higher expression in the PIF sub-region when copared to the PBP sub-region (p < 0.01) (Fig. 3C). Slc17a6 did not show a change in different sub-regions as shown in Fig. 4C. As shown in Fig. 5C,F, GAD1 and GAD2 did not show a change in their expressions in any of the sub-regions.

## Discussion

In our previously published study, we investigated the nicotine-dependent activation of dopamine neurons within the three sub-regions of the VTA (PN, PIF, and PBP) at young adult age group postnatal day 28 (P28) to understand the effect of perinatal nicotine exposure^16^. We tested the specific gene expressions in rat VTA neurons treated perinatally with nicotine. qPCR was used to find the relative quantity of GABA, DA, and glutamate neuron markers and immunofluorescence staining was performed to visualize DA neurons based on their role in the reward/addiction pathway. Our data showed that, following perinatal nicotine exposure, DA neurons are more activated in the PN sub-region of the VTA of young adult age group (P28) rats, as suggested by Ikemoto et al.^17^ After nicotine injection, some VTA DA neurons showed different temporal firing rates^18^. Our results suggest that, within the VTA, there might exist detailed sub-regions of VTA DA neurons, defined by physical function and/or anatomical sub-region.

However, it is important to note that these studies focused on the young adult and adult rats and not on rat pups during early maturation. Therefore, in this longitudinal study during early maturation, we aimed to identify VTA sub-region-specific differences in the expression of dopaminergic and GABAergic neurons, suggesting that these findings may contribute to behavioral differences among the major reward regions of the brain. Therefore, we focused on three sub-regions of the VTA (PN, PIF, and PBP) at three age groups (P7, P14, and P21) to examine the early development of DA neurons in the brain and to identify the effects of early nicotine exposure. When rats are born, the stage of brain development is immature compared to humans. Around gestational day 13 (G13), dopamine neurons of the VTA originate and begin to innervate the neocortex by G15^19^. Dopamine neurons in rats continue to develop and are formed approximately a week before birth (G15–G21)^20^. The first 2 weeks of a rat’s life are comparable to the third trimester of human gestation, and most neuronal cell groups and early synaptogenesis occurs during this stage^13,14^. Dendritic arborization, axonal growth, and gliogenesis also occur during this time^15^. Similar studies showed that perinatal nicotine exposure affects DA release and subsequent DA levels and turnover in the fetal forebrain^4,18,21,22^.

Sub-groups of VTA DA neurons project to different areas of the brain, promoting different responses to nicotine. This indicates different anatomical and physiological functions of VTA DA neurons^18^. Since the VTA also contains GABA and glutamate neurons in addition to DA neurons, markers Slc6a3 and TH were used to detect DA expression, GAD1 and GAD2 for GABA, and Slc17a6 for glutamate. TH is the enzyme that converts tyrosine to l-Dopa for DA synthesis and the most reliable indicator for DA neurons^23,24^. The solute carrier family 6 member 3 (Slc6a3), also known as dopamine transporter (DAT) is another widely used dopamine marker. It regulates the re-uptake of extracellular dopamine into presynaptic neurons and sends dopamine out of the synapse into the cytosol. DAT plays a key role in controlling dopamine levels in the neuron for release. GAD1 and GAD2 play a role in the conversion of glutamate to GABA, the inhibitory neurotransmitter in the central nervous system. Slc17a6, also known as VGluT2, regulates both dopamine transport and l-glutamate transmembrane transporter activity in glutamatergic pathway. Each of these markers have been extensively used to determine the respective neurons. In this study, we have predominantly focused on TH expression as the main marker for DA. However, future studies will also focus more on DAT expression as an indicator of midbrain DA neurons^25^. We hypothesized that the genetic expression of DA neurons will be higher in the nicotine exposed groups as compared to the saline exposed groups of rat pups and that these expressions will become more specific to sub-regions as the rats age^13–15^.

In this study, we found that DA, GABA, and glutamate neurons are present in all three sub-regions of the VTA during brain development from postnatal day 7 to day 21 following prenatal and postnatal nicotine exposure. The main gene expression change happened in TH, DA neurons, in which we observed a consistently increased significance in PN sub-region compared to PIF and PBP at P7 PN vs PBP (p < 0.01), at P14 PN vs PIF (p < 0.05) and PN vs PBP (p < 0.05) and at P21 PN vs PIF (p < 0.05) and PN vs PBP (p < 0.05). This significance in expression of the DA neurons in the PN sub-region of the VTA in the P14 and P21 groups was consistent with our previous study of 28-day old rat pups, where there was a large increase of relative gene expression in DA neurons in the PN sub-region of the VTA compared to the saline group^12^. We also observed that GABA and glutamate neurons were also developing in the sub-regions of the VTA and dominated different regions of the VTA, acting complementary to the development of the DA neurons. These results confirm that the dopaminergic system receives input from a variety of brain regions mediated by local non-dopaminergic neurons. These inputs are functionally important for the regulation of dopaminergic pathways and for neurodevelopment^26^.

It seems increasingly apparent that many, if not all, developing neural circuits display spontaneous activity before they become functionally efficacious^27^. Studies examining the functions of neuronal activity-regulated genes provide compelling evidence for the involvement of this activity-regulated transcriptional program in dendritic outgrowth, synaptic maturation/strengthening, synapse elimination, and synaptic plasticity in adult organisms^28^. The degree to which spontaneous activity instructs developing neural circuits and how activity interacts with gene expression to regulate synapse, neuron, and neural circuit development remains to be a major question in the field^27^.

As a summary, this paper helps us understand the participation of VTA sub-regions in reward and addiction pathways during early maturation. By isolating the sub-regions of the VTA where dopamine neurons are located during early brain development stages, we can create a better physiological map of how the brain is not only developing and responding to the influences of early exposure to addictive substances such as nicotine, but also how we could develop treatments or interventions when necessary. Future studies for these experiments will include detection of the differences between samples of different sexes and observing the pathways leading from each sub-region of the VTA to the PFC. Furthermore, we plan to use in situ hybridization with qPCR in our future studies for a fast and high-throughput detection and quantification of sequences within different matrices.

## Methods

### Animal preparation

All protocols and surgical procedure have been approved by the Institutional Animal Care and Use Committee (IACUC), the University of Houston Animal Care Operations (ACO), were performed in accordance with the accepted guidelines and regulations (protocol code 16-017 and date of approval: July 10, 2019) and were conducted in accordance with ARRIVE guidelines. Pregnant, female Sprague–Dawley rats (Charles River, Wilmington, MA, USA) are ordered to arrive on gestational day 4 and delivered to the University of Houston Animal Care Operations. On gestational day 7, 72 h after arrival, an osmotic pump (Alzet, Cupertino, CA, USA) is implanted subcutaneously containing either nicotine hydrogen tartrate (Sigma-Aldrich, St. Louis, MO, USA) released at a rate of 6 mg/kg/day or an equal volume of saline vehicle for control. Nicotine concentrations implanted in the osmotic pump are calculated based on the weight of the adult rat. Nicotine is then released via osmotic pump and passed on the pups from gestational day 7 to postnatal day 14 during pregnancy and through breastfeeding. Saline filled osmotic pumps are used as control. The animals are observed and weighed daily while kept in the ACO facility with a 12-h light/12-h dark schedule at a temperature of 22 ± 2 °C and 65% humidity. Access to standard food and water was provided.

### Slice preparation

To further understand the neuron distribution and development of DA, GABA, and glutamate neurons following prenatal nicotine exposure in each sub-region of the VTA, samples were taken 7 (P7), 14 (P14) and 21 (P21) days after birth and were compared with saline groups of the same age. On the appropriate postnatal day 7 (nicotine: n = 7; saline: n = 7), day 14 (nicotine: n = 20; saline: n = 14), or 21 (nicotine: n = 18, saline: n = 14), pups were randomly pooled and anesthetized with isoflurane before decapitation. Brains were removed rapidly and sectioned using a VT1200 semiautomatic vibrating blade microtome (Leica, Nussloch, Eisfeld, Germany) at a speed of 0.5 mm/s. Horizontal brain slices of the VTA were obtained by cutting from the ventral side of the brain (1500 µm deep) for ages P7, P14, and P21 before the VTA sub-region samples were collected. For the P7, P14, and P21 groups, 300 µm thick slices were collected for each of the PN, PIF, and PBP sub-regions of the VTA. Two 1 mm biopsy punches (Integra Miltex, VWR, Radnor, PA, USA) were used to collect VTA brain punches bilaterally. Tissue samples were kept fresh in RNAlater (Invitrogen, Thermo Fisher Scientific, USA). Brain slices from each sub-region was taken and samples were collected. Their relative gene expression was analyzed using the primers for qPCR listed in Table 1.

### RNA extraction and cDNA preparation

Total RNA is isolated using Qiagen RNeasy Mini Kit (Qiagen, Hilden, Germany) following pestling of the tissue sample in a RLT buffer and Beta mercaptoethanol solution (Sigma Life Science, Darmstadt, Germany) and sieving the sample through a 20-gauge syringe (Air-Tite Products Co., Inc., Virginia Beach, VA) ten times. Then, cDNA is prepared using High-Capacity cDNA Reverse Transcription Kit (Applied Biosystems, Thermo Fisher Scientific, Carlsbad, CA, USA) according to manufacturer’s instructions and performed on a T100 thermal cycler (Bio-Rad, Hercules, CA, USA) with a final volume of 20 μL following parameters: 25 °C for 10 min, 37 °C for 2 h, 85 °C for 5 min. All reactions were performed in triplicate with a no template control (NTC).

### qPCR

MIQE guidelines were followed to design the qPCR study. Taqman Gene Expression Assay primers, as shown in Table 1, GAD1(Assay ID: Rn00690300_m1) and GAD2 (Assay ID: Rn00561244_m1) for GABA, Slc6a3(Assay ID: Rn00562224_m1) and Th (Assay ID: Rn00562500_m1) for dopamine, and Slc17a6 (Assay ID: Rn00584780_m1) for dopamine and glutamate are performed in triplicate within each sub-region sample. GAPDH (Assay ID: Rn01775763_g1) serves as the reference gene and is performed in triplicate for each sample as well. TaqMan Fast Advanced Master Mix (Thermo Fisher Scientific, Carlsbad, CA) is used with associated assays following parameters: 2 min at 50  °C, 2 min at 95 °C, 40 cycles at 1 s at 95 °C and 20 s at 60 °C on the StepOnePlus Real-Time PCR system (Applied Biosystems, Thermo Fisher Scientific, Carlsbad, CA). Comparative Ct method (ΔΔCt) was calculated using the StepOnePlus Real-Time PCR System, comparing the saline GAPDH sample and used to determine relative expression values. Triplicate RT-qPCR reactions were performed in all validation experiments.

### Statistical analysis

Statistical analysis was performed with GraphPad Prism 8.0 (GraphPad Software, Inc., San Diego, CA, USA), including F test degrees of freedom. Statistical significance was assessed using repeated measures two-way analysis of variance (ANOVA) followed by Tukey’s post-hoc analysis. Unpaired t-tests with Welch’s correction were used when comparing gene expression profiles between nicotine and saline treatment groups in the sub-regions. Statistical information was given as t = t-value, and p = p value. Benjamini–Hochberg False Discovery Rate (FDR) p values were calculated to correct for multiple testing^29^. Genes with FDR adjusted p < 0.05 were considered significantly differentially expressed. Sample sizes were calculated using standard power calculations, requiring an effect size of 30% at 80% power. Values are expressed as the arithmetic mean ± standard error of the mean (SEM). Throughout the manuscript, independent biological replicates are defined as independently performed experiments on material derived from different animals.

### Animal study protocol

The animal study protocol was approved by the Institutional Animal Care and use Committee (IACUC) and the University of Houston Animal Care Operations (ACO) (protocol code 16-017 and date of approval: July 10, 2019).

## Data Availability

Raw data were generated at University of Houston. Derived data supporting the findings of this study are available from the corresponding author Y.M.A on request.
